# "The Hospital" Nursing Mirror

**Published:** 1898-01-15

**Authors:** 


### "The Hospital" Nursing Mirror.

Being the Nursing Section of "The Hospital."

[Contributions for this Section of "The Hospital" should be addressed to the Editor, The Hospital, 28 & 29, Southampton Street, Strand, London, W.C., and should have the word "Nursing" plainly written in left-hand top corner of the envelope.]

### News from the Nursing World.

### AN ENTERTAINMENT IN AID OF THE PRINCESS CHRISTIAN'S NURSES' HOME.

Sir Squire Bancroft is indefatigable in giving his celebrated reading of Dickens's "Christmas Carol" for the benefit of first one and then another charity. He gave it at the Windsor Albert Institute the other day in aid of the Princess Christian's Nurses' Home, and Her Royal Highness was present on the occasion. The room was decorated with palms and flowers from Frogmore, and there was a large gathering. The Dean of Windsor took the chair. He proposed the vote of thanks to Sir Squire Bancroft, which was seconded by the Mayor, and carried unanimously.

### JUNIUS S. MORGAN BENEVOLENT FUND.

All nurses interested in this most excellent and useful fund, so liberally supported by nurses themselves, will be glad to hear that it has received its first legacy. The fund does its work so quietly, and without public appeal, that it is apt to be overlooked when kind-hearted and wealthy people are setting aside their possessions for the benefit of others. An unexpected gift of £250 from the executors of Mr. Richard Gibbs is especially welcome, as showing that the fund is not overlooked in spite of its modest and retiring methods. It must not be forgotten that the very first act in commemoration of the Queen's long reign was that organised by the nurses and their friends on New Year's Day, 1897, when the Princess of Wales received the announcement that £5,000 had been collected as a Jubilee gift to the Junius S. Morgan Benevolent Fund.

### THE GROSVENOR HOSPITAL.

Right glad are the nurses of the Grosvenor Hospital for Women to be comfortably established in their new buildings. The matron has superintended the nursing of the hospital for 18 years, and has seen the rise and progress of many improvements, and is looking forward to other much-needed ones when the money comes in for them. The hospital is now very comfortable, and the electric light is a great convenience. The nurses gain by the good ventilation; whereas, in the old houses that formerly served as the hospital, the heat at night was sometimes so great that profound rest was out of the question, now the even temperature and good air enable them to rise refreshed in the morning. As money is not yet forthcoming to build a nurses' home, the room that will be the nurses' sitting-room is divided into two, and makes comfortable sleeping apartments. The matron's rooms are pretty and comfortable. The baths are of strong enamelled iron, standing on claws, and seem a compromise between the sanitary earthenware baths and the fitted iron baths, as they are easy to keep clean, and do not take so long to get warm as the earthenware. The Princess Louise has sent several pictures nicely framed, which add greatly to the appearance of the wards, and give much satisfaction both to the patients and their nurses. Probationers are trained here for two years, and then receive a certificate, after which they have no difficulty in obtaining the third year's training at a general hospital, as their knowledge of gynæcological nursing is most useful. Candidates not quite strong enough for general nursing at first do well, and matrons sometimes send a promising candidate, whose strength is not equal to the demands of a large institution, to the Grosvenor.

### A LADY OPERATOR.

Miss Marie Corelli, who is recovering from a severe operation, has been throughout her illness under the care of Mrs. Scharlieb, M.D., the well-known surgeon.

### ISOLATION EXTRAORDINARY.

In the dear dark days of not so long ago, if anyone fell sick, the neighbours crowded round in such numbers as seriously to incommode the patient, and effectually to spread the disease. We know better now, with a vengeance. In Inverness-shire a poor woman was smitten with typhoid fever, and was so severely let alone that when some one went—after an interval of some days—to see how she was getting on she was found dead. The sanitary inspector had difficulty in persuading her friends to bury her, and then all they would do was to put the body into a coffin, lift the coffin out of the house, and smear it with a coating of tar. At a place called Delting, a little more than a month ago, a whole family fell sick of scarlet fever, and not one of their neighbours would go near them! Had it not been for the efforts of Miss Turnbull Stewart, who obtained a nurse to wait on them, they must have died. The dread of infectious disease is very rife in the country districts of Inverness-shire and Shetland, and the Zetland County Councillors' endeavour to obtain a trained nurse for every district is a wise and humane one. Though the matter is left over for the present, it seems probable that the Inverness Branch of Queen Victoria's Jubilee Nurses will soon have more applications for nurses than it can supply.

### A NEW CONVALESCENT HOME ON THE RIVIERA.

Authors and artists whose health renders it desirable for them to spend some time in the South have the prospect of a delightful rest at Antibes. Lady Murray has purchased a large house in this neighbourhood, and fitted it up as a home for them. It is about a mile from the railway station, and stands in ten acres of ground. It will be opened this year from February 1st to May 31st, and in future from November 1st to May 31st. Residents must be able to pay their railway fare, personal laundress, and £1 a week. They must also be in a state of health that renders a sojourn abroad desirable.

### EVENTS AT BRADFORD.

The Bishop of Ripon and Mrs. Boyd Carpenter have lately visited Bradford, where his lordship delivered his annual address at St. George's Hall in aid of the joint hospital fund. Before doing so they took the opportunity of going through the wards of the Bradford Royal Infirmary, with which they expressed themselves as much pleased. They were met by some members of the House Committee, and said many kind things both to patients and nurses on their tour of inspection. By a decree of the board held on the 7th inst. the head nurses are in the future to bear the title of "sister," and not "charge nurse" as hitherto.

### SOUTH NORFOLK BENEFIT NURSING ASSOCIATION.

The first annual meeting of this association was recently held at Thetford. It was decided to join the Affiliated Benefit Nursing Associations, and that the several villages should be rated according to population in order to provide a guarantee fund. During the last year three nurses have been placed in districts, and arrangements were made with Garboldisham and Thetford Nursing Associations for the loan of nurses on mutual terms. Subscriptions range from 2s. to 10s. a year, and the payments for the services of a nurse are from 2s. to 10s. a week.

### THE INTERNATIONAL CONGRESS OF HYGIENE AND DEMOGRAPHY.

The ninth International Congress of Hygiene and Demography, which was to have been held last year in Madrid, will take place in that city in April next, from the 10th to the 17th of the month. Ladies who hold a medical or recognised diploma on subjects connected with health will be eligible as members, whilst the wives and relatives of members will be entitled to take part in the social gatherings and excursions upon the payment of a fee of 8s.

### SHOULD GUARDIANS HELP TO SUPPORT THE PARISH NURSE?

The Loughborough Board of Guardians have lately had under consideration an application from the Nursing Association for a subscription. In moving the resolution, Dr. Phelps showed that the nurse had attended seventeen destitute cases, three of which were typhoid, for whom beds and bedding had to be provided by the Association. He proposed that the Guardians should give £20. There was considerable opposition, and the resolution was lost by seven votes to five. Mr. Adcock then gave notice that he would move a resolution that the Board employ a nurse to attend the outdoor paupers. Mr. Phelps remarked that it would cost them £50 instead of £20. This incident raises two interesting points. The first is, should a nursing association make a distinction between the sick poor and sick pauper? The second is, if an Association nurses paupers should not the Guardians help to support it? It seems only reasonable to acknowledge that there is a distinction between the poor and the pauper, for the pauper, being already provided for by the rates, is not a suitable claimant for the charity provided for the poor who are not in receipt of relief. If, then, the Guardians neglect their duty to the sick under their control, the public ought to be made aware of it, for, if they are not, the pauper must suffer until the public insist on the appointment of a proper nurse. When Guardians refuse a £20 subscription to an association which has done their work on the ground that they want to save their ratepayers' money, and at the same meeting give notice of a proposal to spend £50 a year on a private nurse for the whole of whose services they may not have any need, they must expect people to ask where the economy comes in.

### KITCHEN PHYSIC.

The importance of kitchen physic cannot be overrated, so the arts of cooking and serving food are essential to the skilled nurse. Just as attendance on the sick is the only way to teach nursing, so the only place to learn cookery and serving is in the kitchen and pantry. It is most undesirable that food should be eaten either by the hale or sick that has been standing in a living-room, let alone a sick-room; it is contaminated very quickly by impure air, and milk is especially liable to become impregnated with germs. It is also incompatible with economy that the food should be served haphazard. Anyone who has the slightest acquaintance with housekeeping knows that the two chief factors in economy in food are good cooking and dainty serving. A good cook uses every crumb of bread; it is the basis of many delicious puddings and sauces, and the raspings make a necessary adjunct to fried fish, game, &c. Before, however, the cook can do justice to the stale bread, the loaves from which it has been left must have been neatly cut, and the patients must only have been served with pieces that they can eat, and those reserved for cooking must be put in a clean basket or tin. Cooking and carving are both skilled arts, and however desirable it may be—and is—for probationers to learn them in the kitchen, it is quite out of the question to have them taught these things in the sick wards of an infirmary.

### SHORT ITEMS.

Nurse Taylor has completed a very satisfactory year's work at Crediton. The association for which she is the nurse has added £25 to its endowment fund, and begins the new year with a balance in hand of £14.—The Executive Committee of the Barry Victoria Jubilee Home has accepted the revised plan for the proposed building, which is not to cost more than £1,500.—The Nursing Association of Port Glasgow asks increased support, as it is extending the district to which it ministers, and the services of a second nurse are necessary. Nurse Thackery has attended 214 patients during the year, as many as 45 being on the book at the same time.—According to the report of the St. George's Home for District and Private Nurses, Sheffield, there are vacancies for three parish nurses. The year began with a balance in hand of £100, and ended it with one of £7 only. The tramways give the district nurses free passes on their cars, a boon greatly appreciated.—The annual report of the Musselburgh Nursing Association shows a credit balance of £56; the nurse has visited 200 patients during the year.—The Ramsgate District Council has arranged for a series of nine lectures on "Nursing" to be delivered at their offices if a sufficient number of members are forthcoming.—Lady Jeune addressed the annual meeting of the Chieveley and Hermitage Nursing Association. The report was of an encouraging nature, and a fair number of well-wishers of the association were present.—It has been decided at a recent meeting of the Provisional Committee of the County Nursing Association to found a county home for trained nurses at Dorchester, with a branch home at Blandford. Lord Ilchester is the president, Sir Richard Glyn the chairman, and Captain Ackland secretary.—An Italian lady doctor, Dr. Montessori, has been appointed a medical officer of the Red Cross Society.—On the afternoon of January 11th the Duchess of Sutherland gave a concert in the hall of Stafford House, on behalf of Sister Katherine's Home for Nurses at Plaistow. The home realises about £600 by the affair.

### Lectures to Surgical Nurses.

By H. A. LATIMER, M.D. (Dunelm), M.R.C.S. of Eng., L.S.A. of London, Consulting Surgeon, Swansea Hospital; President of the Swansea Medical Society; Lecturer and Examiner of the St. John Ambulance Association, &c.

### XXII.—MODE OF UNION OF FRACTURES—PREPARATION OF PATIENT FOR SURGEON—"CRUTCH PARALYSIS"—SIMPLE ANATOMY OF JOINTS—SPRAINS AND THEIR TREATMENT.

I HAVE told you that compound fractures are very grave; the gravity lies in the fact that the wound having been made in the skin, muscles, and bone, a track is formed for the introduction and development of poisonous germs, and, with the entrance of these germs, we are liable to have the various complications which take place in wounds. The mode of union in simple fractures is similar to that which repairs severe wounds; the irritated blood-vessels in the neighbourhood of the broken bone pour out fluid and white blood cells, and these coagulate and form a glue-like material which sticks the bone together. At first soft and mouldable, after some ten days this glue—called technically "callus"—begins to be converted into bone, and finally, after a varying period, the bone itself is restored to its original condition, even its central canal, in which the marrow of the bone lies, being reformed. When a fracture has been accurately set, and the broken ends are placed in direct contact, little more callus is thrown out than suffices to glue them together. Should there be much displacement, as must occur in such bones as the collar bones and the ribs, which cannot be kept at rest owing to the rise and fall of the chest in the act of breathing, the callus is thrown out around the broken ends of the bone, as well as between it, ensheathing it as in a ferrule; such ensheathing material is known as "provisional callus," and betrays its presence by a lump over the broken bone. Compound fracture are healed by the formation of granulations which fill up the rent in the soft parts.

Few things remain for me to speak of. I dismiss the question of the application of plaster of Paris bandages, and the other varieties of mouldable material, used to keep broken bones in position in place of wooden splints, as you have had practical experience of them in the wards. I must tell you, however, what you shall do when a patient is admitted to the hospital suffering from any of these injuries. Your first duty will be to remove his clothing. Be very careful in doing this; take off the boots very gently, holding the leg with one hand, while you withdraw the boot with the other. Then proceed to take off his clothing; remove trouser, shirt, coat, or other garment at the sound side, and then unclothe the injured one. If you should meet with any difficulty in the latter part of your work, slit the garment up along the seam. Now you will wash the patient, and after having done so, you will carefully dry the injured part, and it is well to dust it with some antiseptic powder, such as boracic powder, for this limits the inconvenience which arises later on, after splints have been applied, by asepticising the perspiration.

When patients begin to move about with crutches they are liable to suffer from what is called "crutch paralysis," a condition showing itself by loss of power and tingling in the hands and arms—feelings akin to the sleepiness of the feet and legs, which you must all have experienced when you have allowed one leg to lie too long over the other when sitting in a chair. This crutch paralysis is due to pressure upon the great nerves of the arm by a badly padded crutch. It is not grave, and is fairly soon recovered from if the use of the crutch is discontinued, but you should know of it, so as to allay the alarm which people feel when they first begin to suffer from it.

After having studied the question of fractures it would seem to be a very appropriate matter to consider the injuries to which joints are liable. I am not going to trouble you with a description of the varieties of joints which are to be met with in the body; it will be sufficient for me to point out that they are those parts of the body where two or more bones join in a manner admitting of motion. The movements allowed to some joints are very slight—to others very free and extensive. Looked at from an anatomical point of view, the parts which form a joint are the ends of the two or more bones which are to be linked together; ligaments of various kinds for keeping these bones in place; gristly or cartilaginous coverings to the ends of the bones to act as buffers in preserving them from violent and injurious shocks; and synovial membranes, to pour out an oily fluid which bathes these different parts and keeps them supple, and so enables them to be freely moved. Any one of these constituent parts of a joint may become inflamed or may be injured, but as a rule several of them become affected together on such occasions.

The commonest injury to which a joint is liable is that known as a sprain. Here, through the operation of some wrench or strain, the affected part has become twisted or turned aside to a greater extent than is allowed by the natural range of movement with which it is endowed; and, during this process of stretching, ligaments are either torn or expanded, and cartilages and membranes are roughly bruised or displaced.

The immediate result of a sprain is pain, heat, and swelling at the injured part, symptoms which mark inflammation as having been set up by the violent disturbance to which the joint has been subjected. The best treatment of such a condition consists in the immediate application of cold to the swollen part, with elevation of the limb so that its blood-supply may be limited. The quicker these measures are adopted after the receipt of the injury the better for the patient, both in relief of suffering and rapidity of cure. Directly after the strain has taken place the patient should lie down and have the raised limb douched with cold water, and after this has been done for some time, and as soon as he feels relieved by it, he should be taken to his home for surgical examination. Do not, as nurses, attempt to ascertain what injury has occurred; leave this matter for the surgeon. Apply cold evaporating lotions until his arrival. Should a dislocation or a fracture have taken place, he will have to deal with them, and you can do no good by handling the joint during his absence, therefore limit your attentions to those measures which ward off or limit inflammation at the seat of injury. The best treatment of sprains, in my opinion, is that of carefully padding the injured joint with cotton-wool and applying a compressive bandage over all. From day to day the bandage is unwound, the wool is removed, and the joint is moved by the surgeon or nurse. This kind of movement is called passive, to distinguish it from active movement, which is the ordinary kind of motion which we indulge in. The treatment which is still too often applied consists in restraint; the joint is enveloped in a plaster of Paris bandage, or some such rigid application, and when this is removed it is found to be stiff and fixed, with some of the muscles which should move it wasted from pressure and disuse, and hot and painful from the existence of a chronic inflammation in its different parts. Such a joint is apt to be a trouble for a long time, and much ill-health may result from the fact that its owner cannot move about to obtain proper exercise to keep himself well. To cure it violent wrenching movements have to be made whereby adhesions are torn and the glue-like lymph which has stuck ligaments, membranes, and muscles together is separated, to allow of free use of the joint; and the limb has to be rubbed to promote free circulation of blood and to restore tone to the muscles. In the majority of cases, if the bandaging and early movement treatment is carried on, these disastrous consequences of sprains may be avoided.

### Post-Graduate Clinics for Nurses.

By a Trained Nurse.

### XL.—THE NURSING OF TYPHOID FEVER (*concluded*).

A few words on the convalescence of typhoid fever will bring the subject to a close. As all nurses know, a general rule exists that a typical typhoid patient remains on fluid diet for at least a week or ten days after the temperature has become normal. At the end of this time, if no other reason exist for care in the diet, a gradual return to solids is made. The temperature should be taken for at least a fortnight after it appears to be running a normal course, and for much longer if any sign of feverishness manifest itself. If the patient has been kept well nourished during the acute stage of his illness his temperature is not nearly so likely to be affected by a return to solid food as it will be if he has been allowed to grow weak through insufficient or unsuitable dieting. Apart from the effect which food has upon the reading of the thermometer, the temperature is often raised by the excitement of sitting up, &c., and the convalescent stage in typhoid—as in so many other illnesses—is too often celebrated by the accession of many congratulatory visitors to the sick room. The contrary custom should prevail of keeping all unwonted visitors from the sick room during the first four or five sitting-up days. For this one excitement at a time is quite enough. In the convalescence of typhoid constipation should be carefully guarded against, and much may be effected by the diet.

Well-made prune jelly—carefully strained so that no skin and little of the prune substance are present—has an excellent effect in causing a little laxity of the bowels, and malt extract is most useful in this direction.

Some debility and anæmia must be looked for in a convalescent typhoid, and a change to the sea or country will often be essential. Here, living as much in the open air as the season and strength permit, the patient should get real rest and good feeding. The culinary resources of many kitchens attached to country and seaside lodgings are often most limited. And for this reason I must always maintain that a nurse is of very little value to an invalid seeking health and recuperation in lodgings unless she can turn her willing hands to cooking and concocting dainty, tempting dishes for her hungry convalescent.

The appetite of a typhoid patient is frequently quite abnormal, and he will feel assured that he can dispose of unlimited quantities of food material But his nurse must sometimes put on a judicious "brake." For long-continued fever weakens digestive power, and typhoid does this more surely than other fevers, involving as it does the gastric as well as the intestinal part of the digestive tract. Food, therefore, while nourishing and plentiful, must be chosen from the standpoint of digestibility. And it must not be forgotten that it is most desirable for a typhoid convalescent to wear a flannel abdominal binder for several months after his recovery from the attack. The wearing of this obviates the sudden and congestive abdominal chills to which many of these patients are liable. The binder can be finally removed without danger, by cutting an inch from the lower part of the binder each morning. Removed thus gradually there is little danger of chill.

Albumenoids are valuable in the feeding of typhoid convalescents as these tend to repair the tissue waste which the long-continued fever has caused. Whipped eggs, oysters, custard puddings, creams, dainty sandwiches, and white fish may be given freely, and some alcohol is usually ordered to assist the unwonted effort of digesting so much food. It should be borne in mind that all the starchy food given to a typhoid convalescent needs prolonged cooking, baking being more effectual than boiling in rendering starchy foods easily digested.

Massage is a great help to many while recovering from a severe type of typhoid, and has a most magic effect in overcoming the emaciation and weakness which in many cases lasts for several months, and occasionally for two or three years. Where convalescence from typhoid drags and halts, a course of massage should always be tried, for it frequently has a marvellous effect in helping the patient to pick up lost health and tone.

### Christmas Keeping.

At the North Staffordshire Infirmary and Eye Hospital many treats were given to the patients. Roast beef, plum pudding, and other dainties were served in the wards at the mid-day dinner, whilst the nurses had their feast in the evening. The Duchess of Sutherland, accompanied by Miss Chaplin, visited the infirmary in the afternoon and brought flowers and books to those patients who could not leave their beds, and afterwards her Grace went to the out-patient department, where a smoking concert was proceeding. She recited Tennyson's "Mother Goose," and then sang, "Don't Cry, Little Girl." On Boxing Day the nurses gave a performance of Mrs. Jarley's waxworks, and on Thursday a very successful Christmas tree was dismantled in the children's ward. The decorations throughout the infirmary were much admired.

Memorial Hospital, Mildmay Park.—On the 30th ult. a happy evening was arranged at this hospital. After a sumptuous tea, a limelight entertainment, lasting for about an hour, was provided by the generosity of some friends of the matron, after which a selection of music filled up the time until Father Christmas, attended by a couple of pages bearing baskets full of gifts, made his appearance, and every one received a memento of the occasion. Of these, Lady Hay, Miss Harward, and other kind friends were the donors.

St. Saviour's Infirmary, East Dulwich.—The Christmas festivities for the patients of this infirmary were concluded on New Year's Day by a tree in the children's ward, hung with gifts. *Truth*, Mrs. Huntingdon, and the girls of the Ady's Road Board School kindly gave the dolls and toys. The elders enjoyed the proverbial good cheer of the season, and Mrs. White generously gave a dramatic entertainment on the Friday following Christmas Day.

The Children's Hospital, Temple Street, Dublin.—On December 30th the patients that could be carried down to the Agnes Ward and those belonging to that ward enjoyed a magic lantern show. All had a very good time at Christmas, the wards were prettily decorated, and there were trees in every one. The toys were chiefly the gifts of children, who took an active part in amusing the little ones by singing and dancing for them.

Sudbury Hall Girls' Home, Wembley.—Miss Chipchase, the matron of the Girls' Home,, has held her post for thirty-one years, and has trained many hundred girls for domestic service. About Christmas time an entertainment is given to the old girls, and many London friends are present as well as the special guests. This year the proceedings were conducted by Mr. Francis Hammond, assisted by Mr. H. Bristow Wallen and Mr. Henry G. Copeland, managing officials of the National Refuges for Homeless and Destitute Children, of which society Sudbury Hall is a branch.

### A Book and its Story.

### THE SINNER.*

THIS is a story with a good plot, in which the characters are skilfully described, and though not "a novel with a purpose," it enforces one of the great lessons of human life. Moreover, it is dedicated to that "great and useful body of workers, the Nursing Sisterhood of Great Britain," and will be perused with keen interest by many of our readers. The story opens in one of the great hospitals of the metropolis, where Nellie Nugent has just arrived to take her place as one of the staff. "The lilac print gown, the white apron, the little cap, gave a touch of Puritanism to the mignonne face and figure. She was a little creature, with an expression of youth and innocence that robbed her of an actual age by several years." As she is nursing in her new surroundings another of the nurses enters, a woman of six or seven and twenty, dark skinned, dark eyed, tall, with a strong, serious-looking face, "a contrast in every way to the small, bright creature before her." It is round these two women that the plot of the story mainly turns. The name of the elder is Deborah Gray, between whom and Nellie Nugent a great friendship arises. Deborah is a woman of a certain force of character—honest, purposeful, and yet with a warm heart. Nellie is of a softer mould, but capable, as appears before the end of the story, of rising to a great emergency. While they are talking during this first interview a patient is carried in—a man, "young, but so wasted and haggard, so dishevelled and ghastly an object, that it was impossible to guess what he was like." He had been brought from a low lodging-house in Westminster, suffering from enteric fever caused by want and neglect. This was Nellie Nugent's first case. She nurses him back to life, and then, when he is convalescent, but still too weak to leave the hospital, they have a conversation, in which she finds out that he is that most miserable of all beings—a social wreck, a gentleman of high birth and education, who has taken to journalism as a profession, but finds there is no market for his wares, the publishers will have nothing to do with his writings. Dick Barrymore is his name, only thirty years old, but feeling already "aged and tired and hopeless." The old story follows. He falls in love with the young girl to whose care he owes his life, and she feels an interest in him, and through her brother, who is a pressman in Dublin, finds him a place on the staff of an Irish paper. But on the very day on which he is to leave the hospital a Deus ex machinâ, in the shape of Geoffrey Masterman, Barrymore's uncle, who has made a fortune in America, appears upon the scene, presents the hospital with a donation of 100 guineas, and carries off the unfortunate Dick, whom he adopts as his son. As "Rita" very truly remarks, "it was altogether so extraordinary, so unprecedented, so much more like the chapter of a novel than the prose of reality." So they part. "In all the world she seemed conscious of nothing but that one empty bed. Mechanically, she went up and smoothed the pillows, her hand lingered over the ivory coverlet. She heard a voice speaking to her, somewhat sharply, 'Strip that bed, nurse, and get it ready, it is sure to be wanted soon.' It was the voice of the head sister. And then Nell remembered she was only a hospital nurse, whose patient had been discharged—cured." Soon after this the authorities of the hospital decide that Miss Nugent is not strong enough to perform the duties of a nurse. She is discharged, and the scene changes to Glengarth, in Ireland, where Nellie Nugent and Deborah Gray are spending a holiday. There, as the experienced novel reader will guess, the two met Dick Barrymore and his uncle. Barrymore is a changed man: well dressed, and bearing in his person all the signs of prosperity; but he is as much in love with Nellie Nugent as ever.

From that point the plot, so far as these two are concerned, unfolds the results of their chance meeting in the London hospital, and whether Mr. Barrymore, who is the most constant of lovers, succeeds or fails, must be learned by reading the story itself. But a new and very tragic element is introduced. Deborah Gray, it seems, has had her own secret in the past, and the man who had blighted her life is staying with his wife at the hotel at Glengarth. He is a doctor, James Langrishe by name; and the nature of his relations with Deborah may be gathered from words which pass between them now. "When I saw you again," he says, "well, it seemed impossible to act the part of a stranger. I felt I must ask you to shake hands and say you'd forgiven me. After all, you were fond of me once." "To my eternal shame and misery," she said. "Now, listen; I am no hypocrite—you know that. If I have a fault, it certainly is not weakness. What wrong you did me, physically and morally, you best know. You slew my faith in all that was good and pure and virtuous. I swore, when I learnt of your treachery, that I would never forgive you. I mean to keep that oath. Now, will you let me pass?"

The man whom the woman thus addressed had thrown her over in order to marry another woman with money; and this lady who is now in bad health has spoken to Nellie Nugent, whom she had heard was a trained nurse, and taken such a fancy to her that she wishes her to come and be her permanent attendant, as much in the character of a friend as a professional nurse. In spite of the language used by Deborah Gray, Dr. Langrishe mentions this to her; but her wrath against the man who has left her for another is so hot that in reply she merely points to Nellie, "My friend is there," she said, with a backward glance; "make your own request to her," and with a sudden quick movement she passed on. The doctor turned to Nellie, who, in the end, became an inmate of the Langrishe household.

The story now turns into a lurid tragedy, in which the centre figure is this Dr. Langrishe, who is "The Sinner." His evil genius is a Lady Ffolliott, who lives in his neighbourhood, and with whom he is on terms of intimacy. Mrs. Langrishe is a poor creature, sickly and unattractive. Lady Ffolliott is beautiful, clever, and unscrupulous. But it would spoil the reader's interest to narrate the sequel; therefore, we leave it here.

This is, as we commenced by saying, a story with a fine plot, in which the characters are skilfully drawn. The scenes in the hospital are well described, with sufficient realism to make them truthful without being of too painful a character. But at one point the writer breaks down and destroys the versimilitude of her work. In many novels the device of introducing a trial scene has been used with much effect. The great master himself, for instance, never wrote anything more pathetic than the trial of Effie Deans in "The Heart of Midlothian." But these are difficult topics to handle. When Mr. Wilkie Collins was writing his "Woman in White" his proof sheets, whenever they related to matters of law, were corrected by a solicitor. And it would have been better had the authoress of "The Sinner" submitted such passages in her book which deal with the intricacies of the law to the legal eye before publication, but with this one, perhaps trivial, shortcoming on "Rita's" part, we heartily commend her story to the public.

* "The Sinner." By Rita. (Hutchinson and Co. 1897.)

### Death in Our Ranks.

WE regret to hear of the death of Nurse Rosina Craney, one of the staff at the Caterham Asylum, Surrey, from pneumonia, at the early age of 20. Her loss will be keenly felt by all her fellow-workers, for her bright and cheerful ways made her a general favourite.

### Everybody's Opinion.

[Correspondence on all subjects is invited, but we cannot in any way be responsible for the opinions expressed by our correspondents. No communication can be entertained if the name and address of the correspondent is not given, as a guarantee of good faith but not necessarily for publication, or unless one side of the paper only is written on.]

### OUR CHRISTMAS DISTRIBUTION.

"The Matron of Westminster Hospital" writes: I wish to express much gratitude to the readers and editor of The Hospital for the beautiful box of clothes received for the patients of this hospital. The things will be most valuable and just what are required for some of the delicate convalescents, who frequently are so very badly provided in this respect.

### A CANCER CASE.

We have received three letters from the nurse who asked for advice about the cancer case, and we are glad to find that our correspondents have given her some useful hints; but she finds her "case" beyond the reach of many of them, and she would be glad to know where oakum may be purchased cheaply. In this week's correspondence there are a number of fresh suggestions which may help her. The correspondence this week will also help her to understand the attitude we take on the subject of a district nurse's pay, and which will, we hope, let her see that the possibility of living on less will not injure the district nurses.

### A DISTRICT NURSE'S PAY.

"E. C." writes: I am pleased to hear that F. Taylor can save 10s. per week out of her salary, but how does she do it? Does she dispense with dinners? Some district nurses seem to do so. Does she ever take a holiday? One would imagine by the tone of her letter that I waste my substance in riotous living. For some time I have helped support an aged parent; death has quite recently made that no longer necessary. Since writing to you I have joined the Pension Fund, but it is quite likely that when the summer comes I shall have to dispense with my usual holiday. I think most people will agree with me that it should be made possible for a nurse to live in comfort, not luxury, now, and to provide for the future without denying herself every little pleasure and making her life one monotonous round of work.

⁂ "E. C." must not allow a feeling of personal irritation to creep into her letters. She has done good by introducing a subject of vital importance for discussion, which if it can be calmly threshed out must be most helpful. If F. Taylor can save 10s. a week out of her very moderate but not unreasonable salary, and will tell us how she does it it must be most instructive, but cannot possibly do harm in reducing salaries, as "E. C." fears. Women trained in nursing can demand reasonable salaries and will not be so foolish as to take less.—Ed. *T.H.*

### NURSE-ATTENDANTS.

"An Invalid" writes: May I suggest through the medium of your widely-circulated paper that a fresh start with the New Year could be made for the supply of nurse-attendants by adding the names of suitable women for this work to the registers of nursing homes? It would supply a great boon to those convalescents who are ill yet not compelled to remain in bed. Trained nurses are expensive and only care to attend serious cases, nurse-companions are needed chiefly for paralysed and mental cases, whereas nurse-attendants could be useful with their needle whilst proving a comfort to a sick person. During the year just closed I have spent £80 in fees alone without counting incidental expenses on sick nurses who could do no needlework, simply from the impossibility of procuring a nurse-attendant who had some hospital training. Such a person would have added greatly to my comfort and been far less costly. I trust that you may agree with my suggestion.

⁂ "An Invalid" might find just the person she wants by advertising for a lady's maid with a good knowledge of sickness. Still there are such attendants to be had, and we print her letter as the best way of introducing her idea of registering them to those whom it may concern. We do not, however, like to see any who are not nurses called *nurse-attendants*.—Ed. *T.H.*

### THE NURSING OF TYPHOID.

The writer of the article criticised writes: Before concluding the subject of the nursing of typhoid fever, I must say a word in regard to the criticisms of "H. B. H.," which appear in The Hospital "Mirror" for January 8th. The writer seems to object to ice-cream chiefly on the somewhat theoretical ground that, as he says, "it must not be swallowed cold." Of course, I need hardly say that if I had thought that it must not be swallowed cold I should never have suggested it. I have many times seen medical men order ice to be actually swallowed in cases of hæmorrhage from the stomach, where surely the "reactive hyperæmia" spoken of by "H. B. H." ought to be still more dangerous; and when one comes to think of it, I should fancy that this objection, founded as it is on the occurrence of "reactive hyperæmia" in the part where there is ulceration, must be rather theoretical, for it is difficult to imagine that the ice cream would arrive at the ulcerated part till long after it had attained the body temperature. I can only appeal to practical experience. But then I do not make ice-creams of "flour or arrowroot." Again, in regard to the so-called koumiss I can but appeal to experience, and I would point out that I specially direct that the koumiss shall be poured "several times from one tumbler to another to expel the air." Nevertheless, although I do not wish to enter on the medical or physiological side of the question, I would ask whether it is not rather straining a point to object to the swallowing of a drink containing pure carbonic acid on the ground that gaseous distension of the bowels must be avoided. One often finds medical men ordering soda water in flatulent conditions, and it seems to me that it is hardly fair to argue about the action of a pure and easily absorbable gas like carbonic acid in the stomach, from data derived from the well recognised evils which accompany the presence in the intestines of the offensive gases of decomposition. The conditions are different. In regard to giving "stimulants all through," of course it must be understood that I only give what the doctor orders. What I have concerned myself about is showing how to give the things ordered in such a way as to settle and do good. Again, although I would not wish to criticise the critic, I think I may fairly call attention to his dictum about yolk of egg. He says, "Yolk of egg is wrong, as it is digested in the duodenum, which has to repair its ulcers, and so has quite enough to do." Of course. But are we to imagine that in the purely milk diet which is the stand by of so many medical men the duodenum is idle? What about the cream? Would "H. B. H." give skimmed milk; and even then does he think that all the milk would be digested in the stomach? If "H. B. H." can pour quantities of cream through his patient's duodenum along with hard curds of milk (which I avoid by my koumiss), why may not I give my patient the yolk of an egg whipped up with brandy? I think physiologists know a great deal about the general principles on which the digestive organs work, but in questions of nice ways of giving things experience has much to say, and I feel sure that medical experience is not at all universally with "H. B. H." in regard to this matter. I think I have seen a mixture called Mist. Vini Gallici given even in typhoid fever.

### THE ROYAL BRITISH NURSES' ASSOCIATION.

Dr. Alderson, late President and Vice-President of the Incorporated Medical Practitioners' Association, writes: I have just read your annotation in last week's "Nursing Mirror," page 123, and it has been pointed out to me that I could give some information to the point in question as I was the first president of the Incorporated Medical Practitioners' Association that had a seat *ex-officio* on the Executive Committee and Council of the R.B.N.A. I was also the year previous to the privilege being conferred by the bye-laws of the R.B.N.A. on the I.M.P.A. a vice-president and a very active member of that association, and was, too, known to the leading members of the R.B.N.A., having been a member of the Council and on its Executive Committee for the one or two previous years 1892 and 1893. It is within my recollection that I attended the meeting convened by the G.P.A. and held at Exeter Hall, when it was agreed *nem. con.* that it would be to the interests of the practitioners and of their society for them to support the Royal British Nurses' Association in their efforts to obtain their charter, but I never heard one word mentioned as to any *quid pro quo* suggested or offered by the R.B.N.A., *i.e.*, such as that the president of the I.M.P.A. should have an *ex-officio* seat in the government of the R.B.N.A., and as I also attended the preliminary council meeting (there was no executive committee in those days)* of the general practitioners, when it was decided to hold the meeting at Exeter Hall, and to which Dr. Bezly Thorne refers. I am therefore able to confirm the accuracy and truth of Dr. Thorne's statement that no such promise had been made, or if so, Why did I not hear of it? My first knowledge of this *ex-officio* seat was some time afterwards when reading in executive committee of R.B.N.A. the draft copy of the bye-laws after the charter had been granted. The distinction of a seat *ex officio* offered to the I.M.P.A. for their President was accepted by me for the society, I being their then President, as a graceful compliment to the general practitioners and to the representative character of their society, and for which the I.M.P.A. expressed their thanks in a letter by their secretary to the R.B.N.A., and this courteous privilege was valued by myself and, as I have reason to know, by my more immediate successors, Dr. Eady and Dr. Jackson, as well as by the Council generally. That this honour for their President has been probably lost to the society I personally regret; but I am in no way surprised, for I know, through, as I think, the mistaken zeal of at least one of their presidents, in the interest, as he thinks, and contrary to the true interests, as I think, of the nurses, has caused the Council and the Executive Committee, more especially of the R.B.N.A., much anxiety and considerable waste of valuable time, and the society expensive and vexatious litigation; and by the publication a few months back by the secretary, on the authority of a small sub-committee, and subsequently adopted as the act of the Central Council of the I.M.P.A. of a document reflecting more or less directly or indirectly on great and honoured names well known as household words by the medical profession, much annoyance to the governing body of the R.B.N.A. I felt this document contained so many unjust and violent reflections, and was published without the knowledge and against the approval of myself and other members of the Council of the I.M.P.A., that I felt compelled to withdraw from the I.M.P.A., a society in which I had held important office and had taken great interest, having been a member *ab initio*, and one of its original founders.

* No executive committee of the I.M.P.A. existed until after the advent of Dr. Bedford Fenwick.

### BED SORES.

WE thank our correspondents for their many interesting papers on the subject of "Bed Sores." So numerous have been the communications which we have received on the subject that we have been compelled to summarise them, and cull from them the most helpful suggestions they contain.

F. TAYLOR nursed two paralytic cases last summer. Under her care the sores of both healed, and when the patients died the skin was sound. Her experience is that nothing is so good as lifting the patient above the moisture on a smooth gutta-percha cushion, hollow in the middle, of a size proportionate to the patient using it. After the sores were healed the backs were kept in good condition by washing, daily rubbing with brandy, and then dusting with zinc powder. The cushions were washed and dried every morning and two mackintosh sheets were kept in use, the one replacing the other every twelve hours. Nurse Taylor draws attention to the fact that charcoal should only be used to clean the sores. She makes her poultices of linseed meal mixed with sufficient charcoal to blacken them. She, with other correspondents, is amused at the idea of our querist objecting to "black, white, or grey work," as in her opinion she "ought not to question the propriety of any order she receives." In this case, as the comfort of the patient was jeopardised by the impossibility of grinding charcoal sufficiently fine by any means at the nurse's command, we think that she was right to call the attention of the medical officer to the character of the charcoal with which she was supplied.

"M. P. T." thinks that the teaching given at Brownlow Hill cannot be improved upon. She recommends zinc powder in preference to starch alone, and says that if, after pounding the charcoal between two sheets of paper with a hammer, it is put into water, it dissolves quickly, and is just as effectual and more cleanly than being sprinkled in the ordinary way.

"A. C. H." recommends the same treatment as the foregoing. She uses circular, ring-shaped pads, made of brown cotton wool covered with old linen, when she cannot get the air cushions. When the skin is slightly broken she paints it with white of egg or collodion. If inflamed or ulcerated, she dresses it with warm boracic lint (not fomentations), renewed every four hours until it is healthy, and then with resin ointment changed three times daily.

"A. M. N." thinks it a pity that our columns should be made the means of instructing inefficient people, and makes some disparaging remarks upon the capabilities of our querist. We would ask her to remember that it is our aim to help all, and that knowledge is always comparative. What is more, this column is set aside to enable those who do know to help those who do not know, and we are glad to find so many willing to use it. That our querist took the trouble to write to us in her difficulty argues that she will take the trouble to learn the best way of grappling with it.

A. TAYLOR is thanked for her letter. She will, however, see that we have already covered the ground she takes.

"M. G. B.'s" article is reserved for consideration.

### Appointments.

### MATRONS.

BELFAST WORKHOUSE INFIRMARY.—Miss Mary Carlile has been appointed Superintendent Nurse of this infirmary. She was trained at Belfast Union Infirmary, and has been working with Queen Victoria's Jubilee Institute, Tadcaster.

THE HOSPITAL, UMTALI.—Miss Blanche Bentham, trained at the Manchester Royal Infirmary, and later night superintendent at the Hospital for Sick Children, Great Ormond Street, has been appointed Matron of the new hospital at Umtali.

BLIND ASYLUM, BRISTOL.—Miss M. Greenhough Smith has been appointed Lady Superintendent at the Blind Asylum, Bristol. Miss Smith was trained at St. Bartholomew's Hospital, and for the last eight years has been matron of the Royal Infirmary, Bristol.

MEMORIAL HOSPITAL, ALMONDSBURY.—Miss M. Moyse Hopkins has been appointed Matron at the Memorial Hospital, Almondsbury. Miss Hopkins was trained at the Royal Infirmary, Bristol, and for the last three years has held the post of out-patient sister.

ISOLATION HOSPITAL, CARNARVON.—Miss L. Owen was on January 4th appointed Nurse-Matron of the above hospital. She was trained at Carlisle, and has since been charge nurse at the Rotherham Union Infirmary and at the Oundle Union Infirmary.

### Minor Appointments.

SMITHDOWN ROAD WORKHOUSE INFIRMARY.—Miss Helen McNaughton has been appointed Charge Nurse of this institution. She was trained at the Borough Hospital, Bootle, Liverpool, and has since been engaged in private nursing.

CENTRAL LONDON SICK ASYLUM, CLEVELAND STREET, W. —Miss Joan Watt, who was trained at the St. Pancras Infirmary, Dartmouth Park Hill, was elected Charge Nurse of this institution on January 3rd, 1898.

### Where to Go.

THE MIDWIVES' INSTITUTE, 12, BUCKINGHAM STREET, STRAND.—On January 24th, at three o'clock, the next course of instruction for midwives will begin. Fee for the full course of twenty-eight lectures, £3 3s. For a single lecture 2s. 6d.

WEST END HOSPITAL FOR DISEASES OF THE NERVOUS SYSTEM, PARALYSIS, AND EPILEPSY, 73, WELBECK STREET, W.—The children's annual entertainment will take place at the hospital on January 15th, at four p.m.

LONDON TEMPERANCE HOSPITAL, HAMPSTEAD ROAD, N.W.—On Friday, at seven, the nurses' entertainment.

### For Reading to the Sick.

"REST in the Lord, and wait patiently for Him."
—*Ps.* xxxvii. 7.

**Verses.**

Why have we yet no great deliverance wrought,
Why have we not truth's banner yet unfurled,
High floating in the face of all the world—
Why do we live and yet accomplish nought?
These are the strivings of unquiet thought,
What time the years pass from us of our youth,
And we unto the Altar of high truth
As yet no worthy offerings have brought.
But now we bid these restless longings cease;
If Heaven has aught for us to do or say,
Our time will come; and we may well hold peace,
When He, till thrice ten years had passed away,
In stillness and in quietness upgrew,
Whose word once spoken should make all things new.
—*Trench.*

Hast thou o'er the clear heaven of thy soul
Seen tempests roll?
Hast thou watched all the hopes thou wouldst have won
Fade one by one?
Wait till the clouds are past, then raise thine eyes
To bluer skies.
Has thou gone sadly through a dreary night,
And found no light,
No guide, no star, to cheer thee through the plain,
No friend, save pain?
Wait, and thy soul shall see, when most forlorn,
Rise a new morn. —*A. Procter.*

If thou could'st trust, poor soul,
In Him Who rules the whole,
Thou would'st find peace and rest:
Wisdom and sight are well, but Trust is best.
—*A. Procter.*

**Reading.**

There is no Peace but being still in God; in always committing all to Him. And they drink the deepest of those refreshing waters who have so far mastered this truth, that He is all to them; that to live is to serve Him. There is a blessed Peace in looking for nothing but our daily task, between this day and the appointed time, when we shall fall asleep in Him. And as this is the secret of peace for this world, so it is for that deeper life of the spirit which we are leading. There is an unspeakable blessedness in knowing that we are in His hands; that He Who created us, that He Who redeemed us, that He Who sanctifieth us, is, indeed, ours in the covenant of this everlasting love. There is a true rest in resigning ourselves to be taught, in yielding ourselves to the leading of His spirit, in coming to prayer, and to worship, and to Holy Communion. and to the daily duties of our station; not as if, through these things, we were to work ourselves up to great attainments, but as that course, in which He, for Christ's sake, will meet us, and lift us up, even to Himself.—*Bishop Wilberforce.*

### Presentations.

MISS ST. CLAIR, the founder and lady superintendent of the St. Laurance Home, Rutland Square, Dublin, the Roman Catholic Branch of Queen Victoria's Jubilee Nurses, was presented with a silver tea service and illuminated address by her nurses on her retirement from active work. Miss St. Clair is a native of Edinburgh, and was trained at the Nightingale School, St. Thomas's Hospital. She intends to reside in Russia.

### Notes and Queries.

**The contents of the Editor's Letter-box have now reached such unwieldy proportions that it has become necessary to establish a hard and fast rule regarding Answers to Correspondents. In future, all questions requiring replies will continue to be answered in this column without any fee. If an answer is required by letter, a fee of half-a-crown must be enclosed with the note containing the enquiry. We are always pleased to help our numerous correspondents to the fullest extent, and we can trust them to sympathise in the overwhelming amount of writing which makes the new rules a necessity. Every communication must be accompanied by the writer's name and address, otherwise it will receive no attention.**

*Edinburgh School for Massage.*

(115) Would you kindly tell me the address of the Edinburgh Training School for Massage, and what fee is charged for the course of training?—*Nurse.*

We cannot find out anything about it. Will anyone kindly give us information?

*L.O.S. Degree.*

(116) Could you kindly tell me where I would receive instructions by correspondence as to the course necessary to pass the L.O.S. examination? I am a private nurse, and wish to study with the idea of taking the L.O.S. degree when I can afford the fee required for practical training.—*Om. Vernon.*

Miss Landport, 52, St. John's Wood Road, N.W., teaches by correspondence.

*Home for Children.*

(117) I should be glad if you would, through your paper, which I take regularly, tell me where I could go (a warm climate and sea air) in England, with three children, two not strong, and live in a small cottage, near to sea, on about £90 a year? I am hoping to train for midwifery, as I have a love for nursing, but am too old for general (being 38). I am the widow of a clergyman, and think if I could let this house, furnished (the furniture is mine) I might be able to live on the proceeds after rent and rates were paid. I have no means, and if I could be trained in midwifery might add to my income. I have excellent recent testimonials, but I find it very difficult with three children to obtain any post. The doctor says the two younger children—aged three and nearly two years —ought to be by the sea in the south.—*Florence.*

We print your letter in full, as some of our correspondents might help you in the matter. We give you also the names of some institutions for the help of the clergy which might assist you either with your training or with the care of your children whilst you were in training. You should, if possible, obtain the London Obstetrical Society's diploma. The Clergy Ladies' Homes, 23, 5, 7, and 29, Formosa Street, Maida Hill, W.; Clergy Orphan Corporation, 62, Lincoln's Inn Fields; the Cholmondeley Charities and the Corporation of the Sons of the Clergy, 2, Bloomsbury-place, W.C.; Bromley College, Kent (pensions and houses, and pensions for clergymen's widows). Apply the secretary. Write stating your circumstances and what you want to do to each.

*Paying Probationer.*

(118) Will you kindly tell me of any hospitals where a lady over forty, but strong and active, could be received as paying probationer? Those preferred which give a certificate after two years' training only.—*L. Z.*

Perhaps the London Hospital, Whitechapel Road, E., would suit you. Write and explain matters to Miss Lückes.

*Testing.*

(119) Would you kindly let me know through your columns of THE HOSPITAL which are the best elementary and advanced books on "urine testing."—*O. and P.*

In practice a nurse can hardly require to know more on this subject than the ordinary methods of making a qualitative examination for albumen and a qualitative examination for sugar. As described in nursing manuals, Refer to "Book World of Medicine" in THE HOSPITAL thisw week.

*Domestics as Probationers.*

(120) Will you kindly tell me if a domestic servant might train for a nurse (hospital). She is 25 years of age, and has excellent six years' reference from present situation as children's maid. Do you think her likely to be accepted? If so, what hospital would be the most suitable, where she might feel happy in her work without being constantly reminded of her inferiority by her fellow nurses. I have read Miss Honnor Morten's book on "How to Become a Hospital Nurse," but cannot gain the necessary information, the servant class not being mentioned. An answer through the medium of your "Nursing Mirror" will greatly oblige.—*Lizzie Verity.*

Suitable candidates of all classes are welcomed by most of the hospitals. Some prefer ladies and some prefer domestics. In whatever hospital you are accepted, however, the treatment you receive will depend chiefly on yourself, Choose your hospital, and send in your application. We should advise you to choose that institution which gives the best training, irrespective of ideas of happiness. Three years soon pass, and a first-rate certificate will counterbalance many of the disadvantages that may attach to lack of gentle birth.

*Lessons in Massage and Cooking.*

(121) *Enquirer* must refer to our rules. She will see then that we only answer queries when the name and address of the sender is enclosed.

**ANSWERS REQUESTED.**

*Case Book.*

(122) Can any nurse give hints as to best method of keeping record of cases in private or hospital nursing?—*F. H.*

*Children's Hospitals.*

(123) Which one of the children's hospitals in London is most in want of funds?—*Stretcher.*

